# Recent Progress on Photoacoustic Imaging Enhanced with Microelectromechanical Systems (MEMS) Technologies

**DOI:** 10.3390/mi9110584

**Published:** 2018-11-08

**Authors:** Changho Lee, Jin Young Kim, Chulhong Kim

**Affiliations:** 1 Department of Nuclear Medicine, Chonnam National University Medical School & Hwasun Hospital, Hwasun 58128, Korea; ch31037@jnu.ac.kr; 2 Departments of Mechanical Engineering, Pohang University of Science and Technology (POSTECH), Pohang 37673, Korea; ronsan@postech.ac.kr; 3 Departments of Creative IT Engineering and Electrical Engineering, Pohang University of Science and Technology (POSTECH), Pohang 37673, Korea

**Keywords:** photoacoustic imaging, microelectromechanical systems (MEMS), MEMS scanning mirror, micromachined US transducer, microring resonator, acoustic delay line

## Abstract

Photoacoustic imaging (PAI) is a new biomedical imaging technology currently in the spotlight providing a hybrid contrast mechanism and excellent spatial resolution in the biological tissues. It has been extensively studied for preclinical and clinical applications taking advantage of its ability to provide anatomical and functional information of live bodies noninvasively. Recently, microelectromechanical systems (MEMS) technologies, particularly actuators and sensors, have contributed to improving the PAI system performance, further expanding the research fields. This review introduces cutting-edge MEMS technologies for PAI and summarizes the recent advances of scanning mirrors and detectors in MEMS.

## 1. Introduction

Photoacoustic imaging (PAI) is a new rapidly growing biomedical imaging tool that is based on the photoacoustic (PA) effect using the configuration of light excitation and the ultrasound (US) capture. It has opto-ultrasound contrast mechanisms and multi-scale imaging ability. Thanks to the characteristics of PA wave generation, PAI enables visualization of relatively deep biological tissues (i.e., from a few millimeters to a few centimeters) as compared to typical pure optical imaging techniques (i.e., up to 1 mm), while maintaining high spatial resolution [1,2,3,4,5,6,7,8,9,10,11]. Also, due to excellent intrinsic optical absorbers in bodies, such as hemoglobin, collagen, melanoma, lipids, etc., PAI provides both anatomical and physiological features. Anatomical features include blood vessels, tendon, melanin, and lipid distributions, and physiological features include hemoglobin concentration, oxygen saturation, blood flow rate, metabolism rates, etc. [12,13,14]. Furthermore, using exogenous contrast agents, PAI can also delineate transparent biological organs, such as lymphatic systems, bladder, intestines, etc. and monitor theranostic process [3,15,16,17,18,19,20,21,22,23,24]. These benefits contribute significantly to basic life sciences and expedite clinical translation in dermatology, oncology, ophthalmology, neurology, etc. [14,25,26,27,28].

PAI systems generally fall into two categories: photoacoustic microscopy (PAM) and photoacoustic computed tomography (PACT), depending on systems performance and hardware configuration [29]. The PAM generates superior spatial resolution using laser or US focusing approach [30,31]. When a small focused laser beam is used to achieve a spatial resolution of the system, the technique is called optical-resolution PAM (OR-PAM). It enables the visualization of microvasculatures in small animals and humans with a resolution of about several micrometers [32,33,34]. Unfortunately, the imaging depth of the OR-PAM cannot be deeper than 1.2 mm due to optical diffusion [13]. When a focused US transducer is utilized to create a high spatial resolution, the technique is called acoustic-resolution PAM (AR-PAM). Even though the US focusing configuration in AR-PAM cannot achieve better spatial resolution than OR-PAM, AR-PAM still provides an enhanced depth penetration of few millimeters. The PACT relies on three-dimensional (3D) reconstruction methods to generate cross-sectional and volumetric PA images. Various types of US transducer arrays are used with improved image acquisition speed for real-time PAI, thus reducing the need for mechanical scanning [35]. Each temporal PA signal of the PA source provides time-resolved and spatially resolved one-dimensional radial data through ultrasonic detection. By combining the temporal and spatial PA data, it is possible to reconstruct a three-dimensional PA image of the source. There are several approaches for determining an optimal reconstruction algorithm that is based on the configuration of US transducer aperture and detection geometry (e.g., planar, cylindrical, or spherical) and are described in [36,37].

These PAI systems with high spatial resolution and multi-scale capabilities are well suited for preclinical applications but they have bottlenecks for clinical translation. In the case of PAM, the rate of image acquisition typically depends on the speed of the scanner. Previous approaches, such as mechanical translation stages and optical galvanometer scanners, have either limited scan speed or low signal sensitivity [13,38,39]. In particular, optical galvanometer scanner can provide high scanning speed in the reflection mode, but it is only valid for unfocused ultrasonic detection configuration because normal optical scanners cannot operate on acoustically coupled media (i.e., water and gel). In the case of the transmission mode, although the optical galvanometer scanner can achieve high sensitivity with the increased speed, the instrumental configuration is not appropriate for clinical use [40]. On the other hand, PACT has limitations in comprehensively visualizing biological organs and tissues, due to several transducer limitations, including limited frequency bandwidth and low sensitivity [41]. For high-frequency transducers, the production process needs thin crystals that are fragile and need complex fabricating process [42]. If a multi-element transducer is used, then a large number of expensive data acquisition (DAQ) are required [43]. For example, a 128 elements transducer array requires 128 DAQs, which is a significant part of the system price. The fabrication of small noise-free transducers for endoscopic and handheld probes is difficult, and they suffer from the shallow field of view (FOV) [44,45].

Microelectromechanical system (MEMS) technology can be a good solution to address these existing PAI challenges. The MEMS-based on micromachining technology has been widely used in industrial and scientific research for more than 30 years [46]. It has several advantages, including size, low weight, low cost with mass production, and excellent performance [47]. Generally, MEMS technology has helped to fabricate functional micro-devices such as sensors, switches, and filters using silicon materials with integrated circuit (IC) fabrication. MEMS technology has also revolutionized several biomedical tools for fabricating miniaturized diagnostic modalities and screening assays, such as micro-sensors, actuators, micro-channels, micro-optics, etc. [48]. These micro-devices based on MEMS technology provide good opportunities to create a new generation of micro-endoscopic and handheld probing systems with the capability of high-resolution in vivo real-time imaging [49,50,51,52,53,54].

In this review, we summarize the current progress of MEMS technologies for PAI and its applications. In Section 2 and Section 3, we briefly introduce the progress made in general silicon MEMS scanning mirrors and the 1- & 2-axis water immersible MEMS scanning mirrors and their applications for PAI systems. In Section 4, we introduce diverse PAI detectors, such as micromachined US transducers (MUTs), microring resonators (MRRs), and micromachined silicon acoustic delay lines and multiplexer.

## 2. Conventional Silicon MEMS Scanning Mirror for PAI

### 2.1. First Generation PAI System Based on MEMS Scanning Mirror

MEMS scanning mirrors have been a major part of current MEMS research [55]. MEMS scanning mirror has a small micro-scale form factor and it has superior scanning characteristics, such as fast and large scanning angle along two axes. Thanks to these advantages, it has been widely used in optical imaging systems, such as optical coherence tomography [56], multiphoton microscopy [57], confocal microscopy [58], head-up display [59], and digital micromirror device (DMD) [60]. In the last decade, MEMS scanning mirrors have been similarly adopted in PAI system to develop small imaging probes for portable applications.

**Figure 1:**
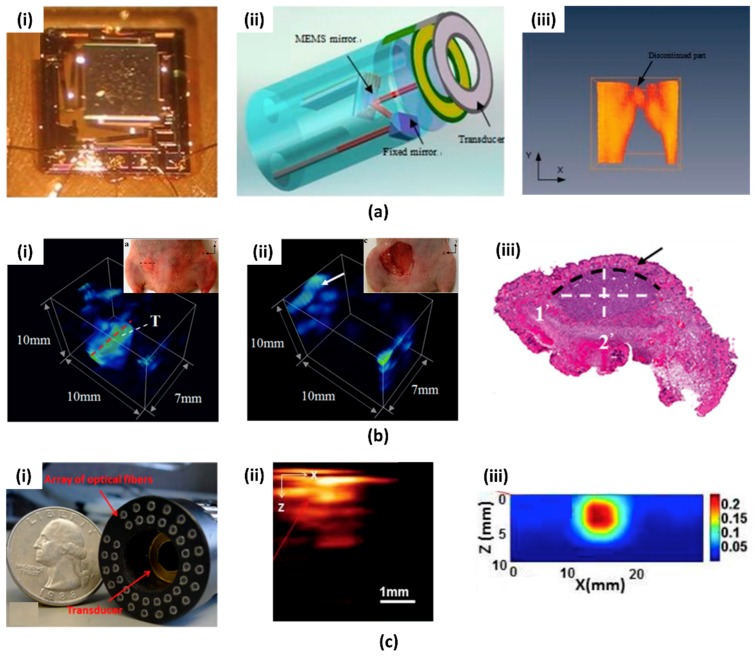
(**a**) Photoacoustic imaging (PAI) probe based on microelectromechanical system (MEMS) scanning mirror (**ai**) Photograph of the MEMS mirror (**aii**) Schematic PAI probe (**aiii**) 3D rendering of the recovered blood vessels of the human hand [61]. Reproduced with permission from Xi, Lei, et al., photoacoustic imaging based on MEMS mirror scanning; published by OSA, 2010. (**b**) In vivo volumetric photoacoustic (PA) image of (**bi**) Tumor before the surgery and (**bii**) the tumor cavity after surgery. (**biii**) H & E stained section along the red dashed line in (**bi**) [62]. Reproduced with permission from Xi, Lei, et al., evaluation of breast tumor margins in vivo with intraoperative photoacoustic imaging; published by OSA, 2012. (**c**): (**ci**) integrated optic fibers and ultrasound transducer probe. (**cii**) Cross-sectional PA image and (**ciii**) optical tomography (DOT) image of the tumor [63]. Reproduced with permission from Yang, Hao, et al., handheld miniature probe integrating diffuse optical tomography with photoacoustic imaging through a MEMS scanning mirror; published by OSA, 2013.

The first MEMS scanning-mirror based PAI system was reported in 2010 [61]. The custom two-dimensional (2D) MEMS scanning mirror developed in this system is shown in Figure 1(ai). A mirror plate was actuated by four electrothermal bimorph-based actuators. As shown in Figure 1(aii), the fabricated MEMS mirror scans unfocused light through the hollow center of the US transducer. Measured lateral and axial resolutions were 0.7 mm and 0.5 mm, respectively. Imaging depths of up to 2.5 mm and an image area of 9 × 9 mm^2^ was achieved. The PAI of pencil lead in chicken tissue and blood vessels in a human hand (Figure 1(aiii)) was demonstrated. Although the MEMS mirror has a scanning speed of up to 500 Hz, the imaging time was slow (i.e., 250 s) because of the slow repetition rate of the laser (i.e., 10 Hz). This MEMS PAI probe was also adopted in intraoperative applications by the same research group [62]. The MEMS imaging probe was updated with high-frequency US transducer for improved spatial resolution and signal to noise ratio (SNR). Using this system, they acquired volumetric PA images of tumor implanted in a live mouse before (Figure 1(bi)) and after (Figure 1(bii)) surgery. Using the obtained PA images, they confirmed the complete resection of tumor post procedure. The size of the tumor matched within 8.5% error margin with the hematoxylin and eosin (H & E) stained sections (Figure 1(biii)). Thanks to the compact design and the performance of the developed MEMS PA probe, it has the potential to be used for image-guided surgery. The MEMS scanning mirror and the PAI system can be integrated with various other optical imaging systems as well. For example, a dual-modality MEMS imaging probe, which integrates PAI with diffuse optical tomography (DOT), was demonstrated in [63]. The MEMS scanning mirror scanned both the pulse laser for PAI and continuous laser for DOT. A ring transducer at the center of probe detects PA signals and optical fibers at the outside of the probe collects diffused light in DOT (Figure 1(ci)). Since the DOT has a lower resolution than PAI (i.e., 3~4 mm), it can be utilized to confirm the position and approximate volume of the tumor (Figure 1(cii)). The PAI with much better resolution (i.e., 0.2~0.7 mm) can be used to display tumor margins accurately (Figure 1(ciii)).

Around the same time, another research group also demonstrated the PAI probe based on a MEMS scanning mirror [64,65]. In their system, they used a commercially available MEMS scanning mirror (TM-2520, Sercalo Microtechnology Ltd., Neuchâtel, Switzerland) to reflect the laser and microring resonator to detect PA signal. These results will be discussed further in a later section.

### 2.2. Recent Advances in PAI System Based on MEMS Scanning Mirror

While the MEMS scanning mirror based PAI probes showed promise, several optimizations were still needed for practical clinical applications, such as (i) increasing imaging speed, (ii) improving spatial resolution and SNR, and (iii) minimizing the probe size for endoscopic applications. Unlike conventional PAI system with external bulky mechanical scanning devices, the MEMS scanning mirror based PAI probes are smaller and faster, while maintaining high-resolution. 

**Figure 2:**
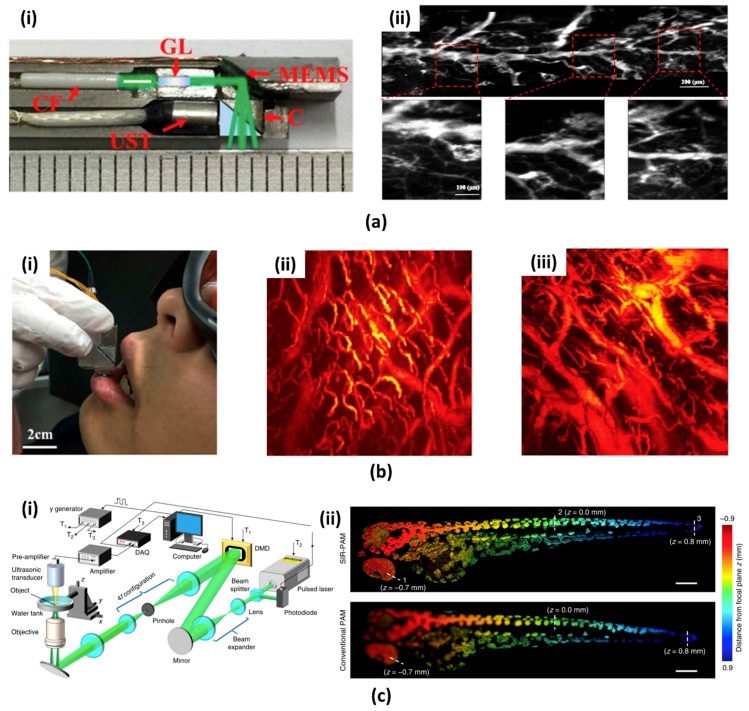
(**a**) MEMS scanning mirror based PA endomicroscopy probe. (**ai**) Photograph of the imaging probe. CF, ceramic ferrule; GL, Gradient-index lens; UST, ultrasound transducer; C, cube. (**aii**) PA image of a mouse colon and sub-images [66]. Reproduced with permission from Guo, Heng, et al., photoacoustic endomicroscopy based on a MEMS scanning mirror; published by OSA, 2017. (**b**) In vivo human oral imaging. (**bi**) Photograph of the PAI probe and a volunteer participating. (**bii**) PA image of the lower lip and (**biii**) back surface of the tongue [67]. Reproduced with permission from Chen, Qian, et al., ultracompact high-resolution photoacoustic microscopy; published by OSA, 2018. (**c**) Digital micromirror device (DMD) based spatially invariant resolution photoacoustic microscopy (PAM) (**ci**) Schematic diagram (**cii**) Depth encoded whole body images of a zebrafish larva. [71]. Reproduced with permission from Yang, Jiamiao, et al., motionless volumetric photoacoustic microscopy with spatially invariant resolution; published by Nature, 2017.

L. Xi group first reported a high-resolution PA endomicroscopy probe using a commercial MEMS scanning mirror (WM-S3.1, WiOTEK, Wuxi, China, commercialized product of Section 2.1) as shown in Figure 2(ai) [66]. This PA endomicroscopy used a 0.7 mm Gradient-index (GRIN) lens to increase the lateral resolution to 10.6 μm. The fast MEMS scanning mirror (i.e., 500 Hz) fully utilized pulse laser’s high repetition rate of 20 kHz. For detecting the PA signals, an unfocused customized US transducer with the axial resolution of ∼105 μm was used. Phantom and animal experiments were demonstrated (Figure 2(aii)) while using this system. Since the diameter of the probe is almost half the size of the previous studies (i.e., 6 mm), it can potentially be used in the endoscopic channel for imaging gastrointestinal tract. Most recently, a MEMS scanning mirror based OR-PAM probe for human lip imaging was developed by the same research group [67,68]. Although the size of the OR-PAM probe is slightly bigger than endomicroscopy, it has better performance with respect to spatial resolution and FOV. The 20 grams weight and 22 × 30 × 13 mm^3^ size of the probe is suitable for imaging the human lip. High lateral resolution of 3.8 μm and FOV of 2 × 2 mm^2^ can provide a PA microvasculature image. This probe was used to image internal organs vasculatures in the rat (Figure 2(bii)) and oral cavity in human (Figure 2(biii)).

The DMD (Discovery 4100, Texas Instruments, Dallas, TX, USA), which consists of several hundred thousand micro mirrors, is another important application of the optical MEMS device. The DMD was also applied in several PAI systems using the spatial and spectral encoding ability of the light [69,70]. Recently, J. Yang et al. reported a motionless volumetric PAM with DMD (Figure 2(ci)) [71]. They used propagation-invariant sinusoidal fringes, by exploiting the field modulation ability of the DMD, for motionless volumetric imaging. The lateral resolution of 1.89 μm that was achieved in this system was 1.5 times higher, and the resolution-invariant axial range of 1800 μm is 30 times higher than the conventional PAM. As shown in Figure 2(cii), they successfully obtained a PA image of zebrafish larva with superior resolution in depth.

## 3. Water Immersible MEMS Scanning Mirror for PAI

### 3.1. 1-Axis Water Immersible MEMS Scanning Mirror

Although the conventional silicon-based MEMS scanning mirror has many advantages in various optical imaging systems, it has one severe drawback for PAI systems. It is that the MEMS fabrication process is generally based on a silicon wafer, which has brittle and delicate mirror supporting structures. Thus, the previous MEMS scanning mirrors are not appropriate to operate in acoustic coupling medium (e.g., water). The PAI systems, as discussed in Section 2, mainly utilized optical beam scanning with unfocused US transducers, which resulted in low detection sensitivity. However, focused detection of PA signals is essential to have high SNR [72] for diagnostic PA images. Conventional PAM makes one dimensional confocal aligning of focused optical and acoustic beams using special components, such as an opto-acoustic beam combiner [13] or ring transducer [73]. For acquiring volumetric images, motor based linear scanning stages are used to move the heavy components, which results in low imaging speed.

**Figure 3:**
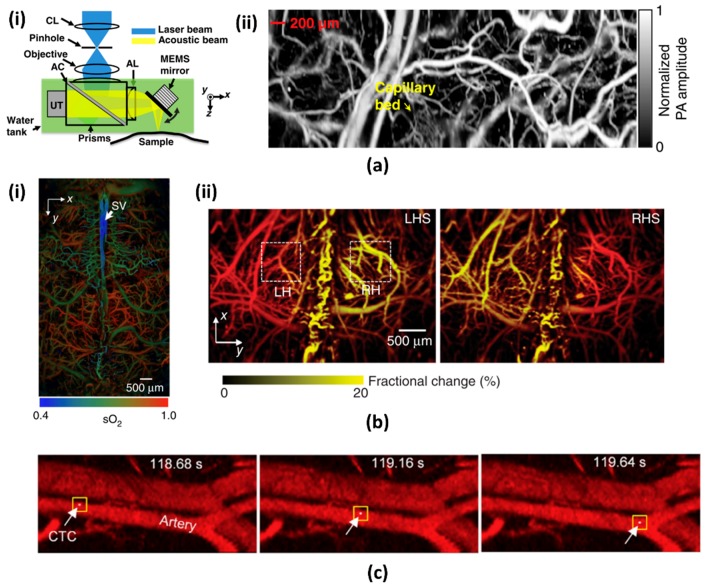
(**a**) A water immersible MEMS mirror based optical-resolution PAM (OR-PAM). (**ai**) Schematic diagram. (**aii**) PA maximum amplitude projection (MAP) image of microvasculature in a mouse ear [74]. Reproduced with permission from Yao, Junjie, et al., wide-field fast-scanning photoacoustic microscopy based on a water-immersible MEMS scanning mirror; published by SPIE, 2012. (**b**) High-speed functional OR-PAM based on MEMS scanning mirror (**bi**) Microvasculatures and oxygen saturation level in a mouse brain. (**bii**) Fractional PA signal changes in response to hindlimb stimulation [75]. Reproduced with permission from Yao, Junjie, et al., high-speed label-free functional photoacoustic microscopy of mouse brain in action; published by Nature, 2015. (**c**) PA snapshots are showing single circulating tumor cells (CTCs) traveling in the vasculature [76]. Reproduced with permission from He, Yun, et al., in vivo label-free photoacoustic flow cytography and on-the-spot laser killing of single circulating melanoma cells; published by Nature, 2016.

To resolve these above-stated problems, J. Yao et al. [74] developed a special 1-axis water immersible MEMS mirror based OR-PAM. The water immersible MEMS mirror used high-strength flexible polymer materials for hinge structures. A mirror plate was made of gold-coated silicon wafer reflecting both optical and acoustic beams. This mirror was actuated by an electromagnetic force between inductor coil and two permanent magnets under the mirror plate. This polymer based hinge structures and high electromagnetic actuation schemes enabled the fast scanning of up to 400 Hz under water. This system greatly enhanced the imaging speed while maintaining high lateral resolution and SNR. Similar to conventional OR-PAM, this system also can utilize the opto-acoustic beam combiner ensuring high SNR. The main difference in this system is that the confocally aligned optical beam and resultant PA wave were simultaneously scanned in water with the fixed beam combiner position (Figure 3(ai)). With fast scanning of the scanning mirror and high repetition rate of the pulse laser, the speed of two-dimensional cross-sectional imaging (i.e., B-scan) was 400 Hz at a wide scanning range (i.e., 3 mm). Additionally, the motorized stage can make a volumetric image by moving the scanning head, MEMS scanning mirror, US transducer, and opto-acoustic beam combiner. Figure 3(aii) shows the PA maximum amplitude projection (MAP) image of the vasculature in a mouse ear over 2 × 5 mm^2^ area. The fast-volumetric imaging rate of 0.8 Hz can show the flow dynamics of hemoglobin in the blood vessels.

A preclinical research study for mouse brain was demonstrated using the high-speed MEMS scanner [75]. Two pulse lasers (i.e., pico- and nano-second pulse), both with a 532 nm single-wavelength, were used to display blood oxygenation with high-resolution. Figure 3(bi) shows the fused PA MAP image of microvasculature and oxygen saturation level in the same mouse brain. The acquisition time for the wide-FOV-mosaic image was about 40 s, which is several hundred times faster than conventional OR-PAM. Figure 3(bii) shows the hemodynamic responses to electrical stimulations in real time. The PA amplitude of right hemisphere was increased in response to electrical stimulation on the left hind limb. The 1-axis water immersible MEMS mirror can also be used in therapy. Y. He et al. demonstrated a PA flow cystography integrated with a laser therapy of melanoma [76]. Similar to the previous results, they first imaged the microvasculature in a mouse ear with a 532 nm wavelength laser. The flow of circulating melanoma cells was acquired while using a 1064 nm wavelength laser. The circulating melanoma cells were immediately killed by another therapy laser, which was self-triggered by the PA signal of the melanoma cells.

### 3.2. 2-Axis Water Immersible MEMS Scanning Mirror

**Figure 4:**
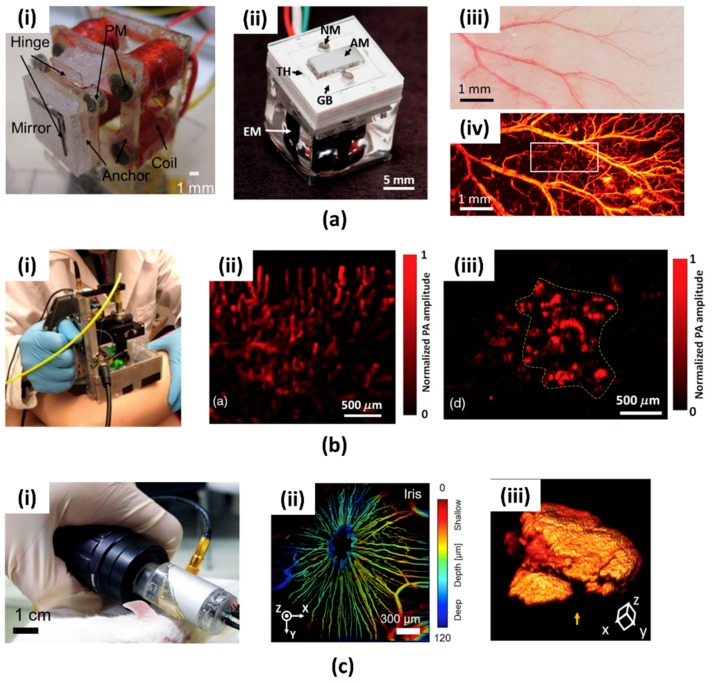
(**a**) 2-axis water immersible MEMS scanning mirror made of (**ai**) biaxially-oriented polyethylene terephthalate (BOPET) film and (**aii**) polydimethylsiloxane (PDMS). (**aiii**) Photograph of a mouse ear and blood micro-vessels in it. (**aiv**) PA MAP image of (**aiii**) [72,77,78]. Reproduced with permission from Huang, Chih-Hsien, et al., a water-immersible 2-axis scanning mirror microsystem for ultrasound andha photoacoustic microscopic imaging applications; published by Springer, 2012. Reproduced with permission from Kim, Jin Young, et al., a PDMS-based 2-axis waterproof scanner for photoacoustic microscopy; published by MDPI, 2015. Fast optical-resolution photoacoustic microscopy using a 2-axis water-proofing MEMS scanner; published by Nature, 2015. (**b**): (**bi**) Photograph of the handheld PAM based on 2-axis water immersible MEMS scanner mirror. (**bii**) PA image of capillaries in a human cuticle. (**biii**) PA image of the red mole on a human leg [80]. Reproduced with permission from Lin, Li, et al., handheld optical-resolution photoacoustic microscopy; published by SPIE, 2016. (**c**): (**ci**) Photograph of the PAM probe. (**cii**) Depth encoded PA image of the microvasculature in a mouse iris. (**ciii**) Volumetric PA image of a mole on a human finger [81]. Reproduced with permission from Park, Kyungjin, et al., handheld photoacoustic microscopy probe; published by Nature, 2017.

As described in the above section, the 1-axis water immersible MEMS scanning mirror can greatly increase the B-scan imaging speed of OR-PAM. However, it still has limitations such as bulky system size due to the additional motorized stage for volumetric imaging. For clinical translation, such as endoscopy, laparoscopy, or handheld systems, it is essential to have both (i) high imaging speed and (ii) small system size. To overcome these limitations, two kinds of 2-axis water immersible MEMS scanning mirror were developed, as shown in Figure 4(ai,aii) [72,77]. Similar to the 1-axis water immersible MEMS scanning mirror, they are also made of flexible polymer instead of brittle silicon. One was fabricated by a laser cutting of biaxially-oriented polyethylene terephthalate (BOPET) film, and the other was made by soft lithography of polydimethylsiloxane (PDMS). They are commonly adapted to a gimbal structure, which can steer the optical and acoustic beam simultaneously along the two axes on one scanner. Aluminum coated silicon mirror enhanced the reflectivity of optical and acoustic beams. Strong electromagnetic actuation along two axes was used to overcome the water resistance.

J. Y. Kim et al. were the first to demonstrate OR-PAM with 2-axis water immersible MEMS scanning mirror [78]. The fabricated 2 axis MEMS scanning mirror that is based on PDMS stamping has a size of 15 × 15 × 15 mm^3^. Without using any motorized stage, this OR-PAM system can achieve a high B-scan rate of 50 Hz and volumetric imaging rate of 0.25 Hz. For this system, lateral and axial resolutions were 3.6 µm and 27.7 µm, respectively. As shown in Figure 4(aiii,aiv), the PA MAP image of a live mouse ear was successfully obtained (Figure 4(aiv)).

Recently, this 2-axis MEMS scanning mirror was commercialized by Opticho Inc., Ltd. in South Korea. M. Moothanchery et al. reported an OR-PAM system using this commercial 2-axis water immersible scanning mirror [79]. This system shows a high lateral resolution of 3.5 µm in spite of using multimode fiber.

L. Lin et al. demonstrated a handheld PAM system based on 2-axis water immersible MEMS scanning mirror, as shown in Figure 4(bi) [80]. This handheld OR-PAM system has a dimension of 80 × 115 × 150 mm^3^ and is more flexible than conventional benchtop systems. The lateral resolution of the handheld system was 5 µm. The 3D volumetric imaging rate over a region of 2.5 × 2.0 × 0.5 mm^3^ was 2 Hz. To verify the usage of the handheld PAM in clinical applications, they acquired PA images of human cuticle (Figure 4(bii)) and a mole on a volunteer’s leg (Figure 4(biii)). K. Park et al. reported a much smaller handheld OR-PAM probe, as shown in Figure 4(ci) [81]. They modified the water immersible MEMS scanning mirror to a round shape to reduce the system size. All of the parts, including 2-axis MEMS scanning mirror, were integrated into this small probe (diameter: 17 mm). The lateral resolution was 16 µm, and the B-scan rate was 35 Hz. Thanks to the small size and fast imaging speed, this handheld probe is suitable for both small animal and human imaging. Figure 4(cii) shows the in vivo depth encoded microvasculature image of the mouse iris and Figure 4(ciii) shows the 3D image of a mole on a volunteer’s finger. In Table 1, we summarize and present the specifications of all MEMS scanning mirrors compared to conventional scanning methods (i.e., mechanical stage and optical galvanometer scanner).

## 4. Micromachined US Detector for PAI

### 4.1. Micromachined US Transducers (MUTs)

Transducers arrays made of polyvinylidene fluoride (PVDF) have been widely used in clinical PAI systems [82,83]. However, due to the relatively low sensitivity and limited frequency bandwidth of small PVDF, there are limits to using mini-sized probes for endoscopic and vascular applications. MUTs can be an excellent alternative to overcome these issues with broad frequency bandwidth and miniaturized size. MUTs are divided into two types: capacitive MUT (CMUT) and piezoelectric MUT (PMUT). 

**Figure 5:**
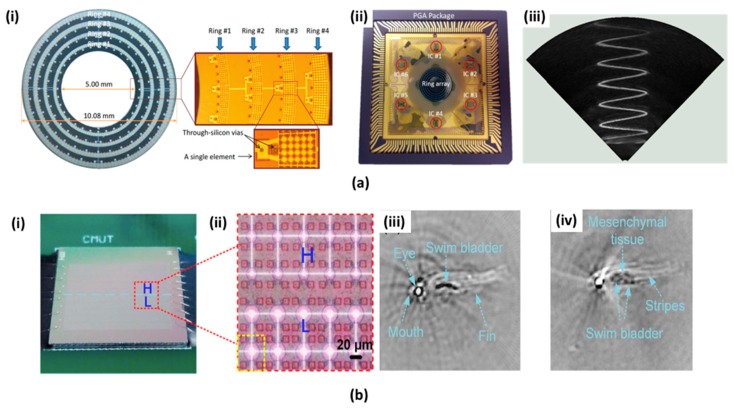

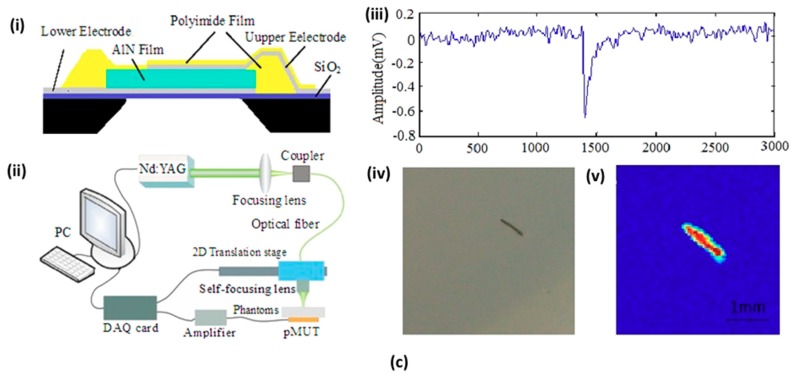
(**a**) A ring-type capacitive micromachined US transducers (CMUT) array for PAI. (**ai**) Schematic and an optical microscopic view of the 512-element four-ring CMUT array. (**aii**) A ring CMUT array installed into handmade electronics. (**aiii**) Volumetric US image of a metal spring with the ring CMUT array [88]. Reproduced with permission from Nikoozadeh, Amin, et al., an integrated Ring CMUT array for endoscopic ultrasound and photoacoustic imaging; published by IEEE, 2013. (**b**) Multi-band CMUT array for PAI (**bi**) The photograph of multi-band CMUT array. (**bii**) Magnified optical microscopic view of multi-band CMUT array. (**biii**,**biv**) In vivo PA images of a zebrafish measured by the low- and high-frequency CMUT arrays, respectively [90]. Reproduced with permission from Zhang, Jian, et al., development of a multi-band photoacoustic tomography imaging system based on a capacitive micromachined ultrasonic transducer array; published by OSA, 2017. (**c**) A PMUT based on AlN and acquired PA image (**ci**) Structure of PMUT based on AlN. (**cii**) Experimental setup of photoacoustic imaging with PMUT. (**ciii**) Acquired PA signal of the hair (**civ**) Photography of human hair embedded in the phantom. (**cv**) Reconstructed PA image [98]. Reproduced with permission from Chen, Bingzhang, et al., AlN-based piezoelectric micromachined ultrasonic transducer for photoacoustic imaging; published by AIP, 2013.

CMUT utilizes capacitance variation that is related to energy transduction between a silicon substrate and a thin membrane layer to detect the US signal. It has several unique advantages, such as (i) convenient interfacing with front-end electronic circuits and (ii) can be easily manufactured to have diverse array sizes with individually linked electronics [84,85,86,87]. This technology has already been applied in compact two & three-dimensional US and PA handheld and endomicroscopic probes. As shown in Figure 5(ai), A. Nikoozadeh et al. reported a ring-type CMUT array that comprised of four concentric rings fabricated with a polysilicon sacrificial release process [88]. All concentric rings were located in the main probe body with different diameters (i.e., 6.0, 7.2, 8.5, and 9.7 mm). Same 128 transducer elements were used at each concentric ring with different center frequencies (i.e., 16, 12, 8, and 6.5 MHz). The probe has an inner diameter of 5.0 mm and the outer diameter of 10.1 mm. The miniaturization provides a good opportunity to use them in endoscopic PAI systems. To reduce general loss and improve SNR, the ring CMUT arrays were installed into a handcrafted IC in a pin-grid-array (PGA) and was fully connected to commercial PAI systems (Figure 5(aii)). A 128-channel US imaging platform (Verasonics, Inc., Redmond, USA) was used to receive PA waves. In Figure 5(aiii), the volumetric image of the metal spring was obtained by 360 degrees rotation of the B-mode plane along the vertical axis and accumulating the MAP. J. Chen et al. developed an infrared-transparent silicon CMUT array that provides a compact probe size and uniform laser excitation configurations [89].

A multi-band CMUT was also fabricated by J. Zhang et al. to visualize the more comprehensive structure of biological tissues [90]. Figure 5(bi,bii) show the photographs and the magnified optical microscopic image of the multi-band CMUT array comprising of low-frequency (~4 MHz central frequency, 10 µm radius) and high-frequency (~10 MHz central frequency, 15 µm radius) arrays. To fabricate the CMUT array, four-inch silicon wafer consisting of the substrate and a lower electrode was prepared. The CMUT and channels were fabricated through a reactive-ion etching (RIE) process with the polysilicon layer and deposition of the Si_3_N_4_ layer. Finally, a thin film aluminum of 300 nm was deposited to fabricate an electrode, a connection portion, and a bonding pad. Figure 5b(iii,iv) show the in vivo PA images of zebrafish that were obtained with the multiband CMUT.

PMUT is also an emerging US detector based on flexural vibration induced by a thin-film piezoelectric membrane. The PMUT provides different benefits compared to CMUT including (i) relatively higher capacitance as compared to CMUT, (ii) does not require high polarization voltage, and (iii) has a compatible matching impedance with sample [91,92,93]. Several types of PMUT-based US transducers are widely used for biomedical applications, such as the catheter type, dome-shape array, and concave array type [94,95,96]. W. Liao et al. first reported the two-dimensional PMUT array with 144 elements for the PAI [97] system. They developed a PMUT array by fabricating a thin film PZT membranes with a radius of 25 μm and a pitch of 80 μm. The membrane consists of a PZT layer of 0.6 μm, an elastic SiO_2_ layer of 1 μm, and covering layer of 5 μm. In the pulse-echo mode, the high resonant frequency of 10 MHz, good spatial gain, and broad capturing angle have been demonstrated.

B. Chen et al. proposed a new PMUT based on an aluminum nitride (AlN) for PAI applications [98]. As shown in Figure 5(ci), the proposed PMUT has micro-layers comprising of SiO_2_, lower/top electrodes, AlN, and polyimide (PI) films. The piezoelectric layer based on AlN was generated from metallic Al samples at room temperature via intermediate frequency magnetron reactive sputtering. The AlN based PMUT shows the enhanced SNR due to AlN’s relatively low piezoelectric coefficient. Additionally, its manufacturing process has the benefit of being compatible with the standard ICs. Figure 5(cii) shows the schematic of the PAI experimental setup. A nano-second pulsed laser with 532 nm (Brilliant, QUANTEL) wavelength and was coupled to a mulimodal fiber was excited inside this phantom. The generated PA signal was identified by the PMUT array that was located at the bottom of the phantom. Figure 5(ciii,civ,cv) show one-dimensional PA signal, sample photographs, and reconstructed PA image of human hair within the phantom, respectively. The measured lateral resolution was 240 μm.

### 4.2. Microring Resonators (MRRs) 

Typically, a conventional piezoelectric US transducer works in the resonant frequency band, which is determined by the thickness of the piezoelectric crystal. When a thin piezoelectric crystal film is used to produce a high-frequency transducer, this thin film is fragile and it causes manufacturing complexity and ruggedness issues [42]. Also, these transducers have a low axial resolution because of limited bandwidth and have small FOV because of limited capturing angle. They are also difficult to integrate with high-resolution optical microscopy, which has short working distance (i.e., below 1 mm) [90,99]. To address these drawbacks, diverse optical based ultrasonic detection methods, such as Fabry-Perot polymer film [100], Michelson interferometer [101], Mach Zehnder interferometer [102], and MRR [52,103,104,105] have been reported with an easy-to-apply configuration for endoscopic and microscopic systems and superior US sensing capability. Among these approaches, MRR has additional strengths. (i) A sub-millimeter sized MRR enables high US sensitivity, which reduces the optical interference in probing configuration. (ii) Broadband ultrasonic wave detection can be achieved in MRR, which enhances the axial resolution in PAI and ultrasonic imaging (USI). (iii) The MRR detector allows for a relatively high ultrasonic detection angle, which improves the FOV.

**Figure 6:**
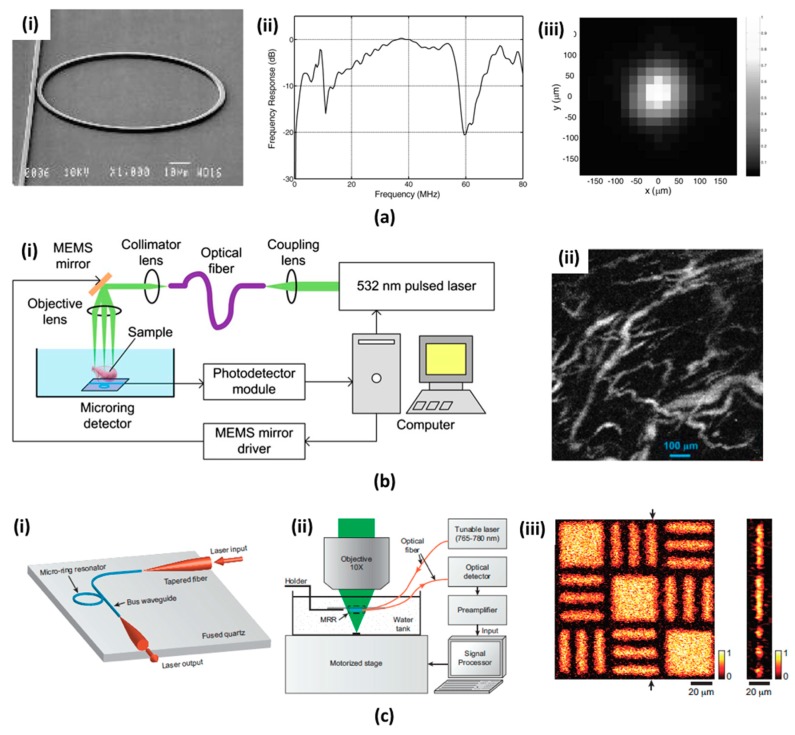
(**a**) Geometry of micro-ring resonator (MRR) US detector and measured properties (**ai**) Scanning electron micrograph of MRR. (**aii**) The frequency response of MRR to a US pulse. (**aiii**) Two-dimensional US pulse response of MRR [52] Reproduced with permission from Chao, Chung-Yen, et al., high-frequency ultrasound sensors using polymer microring resonators; published by IEEE, 2007. (**b**) Miniaturized OR-PAM using MRR (**bi**) Schematic of optical-resolution PAM system using the MRR. (**bii**) MAP image of the microvasculature in the mouse bladder [64]. Reproduced with permission from Chen, Sung-Liang, et al., miniaturized all-optical photoacoustic microscopy based on microelectromechanical systems mirror scanning; published by OSA, 2012. (**c**) Transparent broadband MRR US detector for OR-PAM. (**ci**) Schematic of MRR US detector with tapered optical fibers. (**cii**) Experimental setup for OR-PAM with transparent MRR. (**ciii**) The MAP image of a carbon-black thin film sample along an x-y plate and a two-dimensional PA image of the target at the location indicated by the arrows [108]. Reproduced with permission from Li, Hao, et al., a transparent broadband ultrasonic detector based on an optical micro-ring resonator for photoacoustic microscopy; published by Nature, 2015.

C.-Y. Chao et al. first reported a polymer MRR detector as a US transducer [52]. It was designed in such a way that the ring and the straight-line bus waveguides were interconnected (Figure 6(ai)). They used polystyrene (PS) as the waveguide material, which has the advantages of high sensitivity for acoustic pressure and low absorption for visible to near IR spectrum light. The width of the waveguide is 2.4 μm and the height is 1.85 μm. Nanoimprint process was applied to fabricate waveguides with high sensitivity. First, a mold with an inverted pattern was produced using electron beam lithography and RIE. Subsequently, a spin-coated polymer was imprinted onto the substrate by using the fabricated mold at an appropriately increased temperature and pressure. By applying pulse-echo signals, the MRR response was acquired, as shown in Figure 6a(ii). The bandwidth increased by 10 dB from 15 MHz to 58 MHz and decreased approximately from 60 MHz onwards. The active imaging area was investigated with two-dimensional US emission on the surface of MRR. The measured signal has the full width at half maximum (FWHM) of approximately 130 μm (Figure 6(aiii)). C. Zhang et al. upgraded the polymer bandwidth from dc to 350 MHz, which presented the outstanding axial resolution of 3 μm [106].

The concept of miniaturized PAI and all optical customized PAI were successfully demonstrated based on MRR’s advantages [64,103,107]. For instance, S.-L. Chen et al. reported the miniaturized PAM probe with MRR detector and MEMS optical scanning mirror. Figure 6(bi) shows the system configuration. A high speed diode-pumped solid-state Nd:YAG laser at 532 nm was directly inserted into an optical fiber and was transferred to a 2-axis MEMS scanning mirror. The MRR detector was located 3.7 mm below the sample surface. System performance was demonstrated by visualizing the microvessels in a mouse bladder with lateral and axial resolutions of 17.5 μm and 20 μm, respectively. Even though these MRRs were investigated on silicon plates, they are not optically transparent. Therefore, only permeable PAI system configurations that are not suitable for scanning thin layered samples were possible. H. Li et al. fabricated an optically transparent US detector that was composed of the MRR on a fine coverslip [108]. Figure 6(ci) shows the customized MRR US detector. The two tapered optical fibers combined with the input and output stages of the ring and bus waveguide simplify the packaging process and improve the coupling efficiency. Due to the optical transparency of the MRR detector, a highly focused laser beam was irradiated on the thin samples via the MRR detector located on the adjustable holder. When compared to the transmission PAI configuration, US deformation was eliminated. The developed MRR US detector has an ultra-wideband frequency range (approx. 140 MHz) and provides an excellent axial resolution of 5.3 μm. A thin film sample was used to obtain a PA image with improved axial resolution and is shown in Figure 6(ciii)).

### 4.3. Micromachined Silicon Acoustic Delay Lines and Multiplexer 

**Figure 7:**
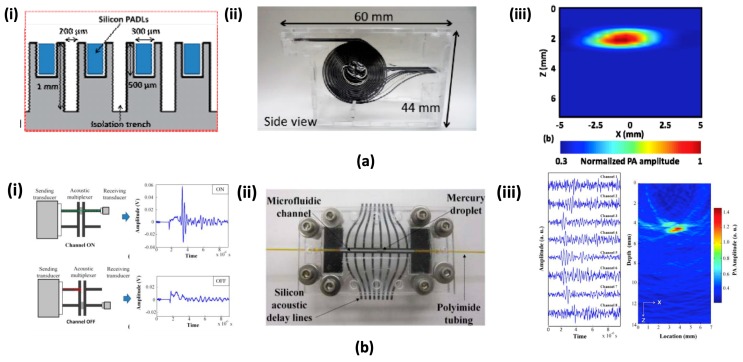
(**a**) Micromachined parallel silicon delay lines. (**ai**) An enlarged view of spacers with detail dimension. (**aii**) Fabricated 16 channels parallel delay lines and assembled on an acrylic housing. (**aiii**) Reconstructed two-dimensional PA image of the absorber in the phantom with 16-channel parallel delay lines [112]. Reproduced with permission from Cho, Young, et al., a micromachined silicon parallel acoustic delay line (PADL) array for real-time photoacoustic tomography (PAT); published by SPIE, 2015. (**b**) Micromachined acoustic multiplexer (**bi**) Acoustic ON/OFF characterization. (**bii**) An assembled acoustic multiplexer (**biii**) Photoacoustic signal and reconstructed image [113]. Reproduced with permission from Chang, Cheng-Chung, et al., a micromachined acoustic multiplexer for ultrasound and photoacoustic imaging applications; published by IEEE, 2014.

Typical array-type US transducers used in clinical USI and PAI require multiple complex multi-channel DAQ devices to simultaneously receive large amounts of acoustic data from each transducer element [109]. This increases the overall PAI complexity and cost of the system. Recently, the concepts of acoustic time delay were reported by M. K. Yapici et al. [110] to reduce complexity. The parallel connected acoustic delay line receivers were utilized instead of the transducer elements. Each delay line detected the acoustic signal and generated an appropriate delay time so that the signal arrived at a different time on the other side. A single transducer was connected on the opposite side for sensing the time delay signal in series. Thus, the delay line reduces the requirements for multi-element transducer elements and multi-channel DAQ devices. This approach would be more cost effective than conventional US detecting systems. The handheld optical fiber based delay line was investigated as a promising method to take several advantages, such as less acoustic loss, microscale size, flexible property, and low cost [111]. However, in order to generate enough time delay in the optical fiber, a considerable length of optical fiber is required due to the high US velocity in the medium. Moreover, additional attenuation and signal distortion could also occur due to the covered jacket layer. Optimal optical alignment is also necessary to obtain a proper signal and manual assembly. Y. Cho et al. introduced a micromachined silicon acoustic delay line [112]. Thanks to the material property of single crystalline silicon, this method has better transmission efficiency, small size, and more productivity when compared to the optical fiber-based delay lines. Each acoustic channel delivers a single acoustic signal with a specific travel path and delay. To generate sufficient delay length and maintain a compact size, each acoustic channel consists of several U-turns. As shown in Figure 7(ai,aii), 16-channel parallel lines were fabricated by an RIE process using the aluminum pattern mask. All fabricated delay lines were located on the acrylic housing. Since the ultrasonic pulses propagate different lengths from the delay line, they reached the outputs at different times. Figure 7a(iii) shows the acquired two-dimensional PA image from the proposed parallel delay lines. A similar concept was adopted by the same group to micromachined acoustic multiplexer [113]. Only one transmit and/or receive US transducer was required to resolve multichannel signals in this system. Unlike the acoustic delay line, acoustic multiplexer can selectively transmit the acoustic signal via the movement of mercury droplet in microfluidic channel (Figure 7(bi)). The assembled multiplexer is shown in Figure 7(bii). The silicon delay line and multiplexer structure were fabricated by the RIE process. Two PDMS sealing pads were used to form a microchannel with the silicon structure, and the PI microtubing was connected to inlet and outlet of the channel. Mercury droplet was driven by a syringe pump. The PA image of the phantom using this system is shown in Figure 7(biii). A pulse laser illuminated a 5 × 5 mm^2^ area and the PA signal generated was successfully detected by a single transducer. To collect eight channel signal, illumination and acquisition were repeated eight times.

## 5. Conclusions

In this review, the current progress of PAI based on MEMS technology was presented. From the MEMS scanning mirrors perspective, they have shown several advantages, including fast scanning abilities, compact sizes, and high SNRs. In particular, the water immersible MEMS scanning mirrors broke through the intrinsic limitation of PAM techniques that were caused by acoustic coupling medium (i.e., water). New advances also contributed to the fabrication of the well-established preclinical PA handheld probes and PA endoscopic systems for brain studies, angiogenesis, and cancer studies. From the MEMS detectors perspective, diverse PA detectors, such as MUTs, MRRs, and acoustic delay lines were introduced. MUTs enable wide frequency bandwidth, small size, and conventional integrating process with electronics. These contribute to develop a multispectral clinical PA system with endoscopic or handheld probes. Similarly, MRRs have excellent performance in the wide frequency band, enhanced FOV, and high sensitivity. Especially, because of its micro-scale resolution, this can also be applied to PA endoscopic and microscopic imaging systems. Acoustic delay lines show the potential for a new cost-effective acoustic delivery and mixing tool. In spite of these advances in MEMS technology, further optimizations are needed for clinical use. First, the currently developed water immersible MEMS scanning mirrors are not yet micro size, which limits their application for endoscopic type device. There is also a need to reduce scales, such as t that of silicon-based MEMS scanning mirror through the development of advanced microfabrication. In addition to the MEMS scanning mirror, MUTs, MRR, and acoustic delay liens, also require special and expensive fabrication process, such as e-beam lithography and anisotropic etching with high aspect ratio. These fabrication processes make it difficult to achieve mass production and stable system performance. Thus, there is a need to develop simple microfabrication process to reduce cost as well as to increase reliability. If these challenges are resolved, we expect the MEMS technologies to contribute greatly to the development of high-performance and clinically useful PAI systems.

## Figures and Tables

**Table 1:** Comparison of the PAI system based on MEMS scanning mirror.

Scanning Methods	System Size	Imaging Speed (B-Scan Rate)	FOV	SNR	Ref.
Mechanical Scanning	Bulky (>300 mm)	1 Hz	>10 mm	+++	[12]
Optical Scanning	Bulky (>200 mm)	100 Hz	<8 mm	+	[39]
Silicon MEMS Mirror	Small (<30 mm)	500 Hz	<3 mm	++	[67]
Water immersible MEMS Mirror	Medium (<100 mm)	1 axis: 400 Hz 2 axis: 50 Hz	<3 mm <9 mm	+++	[75,78]
Handheld PAI Probe	Small (<30 mm)	35 Hz	<2 mm	+++	[81]
